# Incorporating machine learning and social determinants of health indicators into prospective risk adjustment for health plan payments

**DOI:** 10.1186/s12889-020-08735-0

**Published:** 2020-05-01

**Authors:** Jeremy A. Irvin, Andrew A. Kondrich, Michael Ko, Pranav Rajpurkar, Behzad Haghgoo, Bruce E. Landon, Robert L. Phillips, Stephen Petterson, Andrew Y. Ng, Sanjay Basu

**Affiliations:** 1 Department of Computer Science, Stanford University, 353 Serra Mall, Stanford, CA 94305 USA; 2 Department of Statistics, Stanford University, Stanford, USA; 3 Department of Healthcare Policy, Harvard Medical School, Boston, USA; 4 Center for Primary Care, Harvard Medical School, Boston, USA; 5 Center for Professionalism & Value in Health Care, American Board of Family Medicine Foundation, Lexington, USA; 6 Robert Graham Center, American Academy of Family Physicians, Leawood, USA; 7 Research and Analytics, Collective Health, San Francisco, USA; 8 School of Public Health, Imperial College London, London, England

**Keywords:** Risk estimation, Machine learning, Social determinants of health

## Abstract

**Background:**

Risk adjustment models are employed to prevent adverse selection, anticipate budgetary reserve needs, and offer care management services to high-risk individuals. We aimed to address two unknowns about risk adjustment: whether machine learning (ML) and inclusion of social determinants of health (SDH) indicators improve prospective risk adjustment for health plan payments.

**Methods:**

We employed a 2-by-2 factorial design comparing: (i) linear regression versus ML (gradient boosting) and (ii) demographics and diagnostic codes alone, versus additional ZIP code-level SDH indicators. Healthcare claims from privately-insured US adults (2016–2017), and Census data were used for analysis. Data from 1.02 million adults were used for derivation, and data from 0.26 million to assess performance. Model performance was measured using coefficient of determination (R^2^), discrimination (C-statistic), and mean absolute error (MAE) for the overall population, and predictive ratio and net compensation for vulnerable subgroups. We provide 95% confidence intervals (CI) around each performance measure.

**Results:**

Linear regression without SDH indicators achieved moderate determination (R^2^ 0.327, 95% CI: 0.300, 0.353), error ($6992; 95% CI: $6889, $7094), and discrimination (C-statistic 0.703; 95% CI: 0.701, 0.705). ML without SDH indicators improved all metrics (R^2^ 0.388; 95% CI: 0.357, 0.420; error $6637; 95% CI: $6539, $6735; C-statistic 0.717; 95% CI: 0.715, 0.718), reducing misestimation of cost by $3.5 M per 10,000 members. Among people living in areas with high poverty, high wealth inequality, or high prevalence of uninsured, SDH indicators reduced underestimation of cost, improving the predictive ratio by 3% (~$200/person/year).

**Conclusions:**

ML improved risk adjustment models and the incorporation of SDH indicators reduced underpayment in several vulnerable populations.

## Background

Public and private regulators use risk adjustment models to prevent adverse selection, anticipate budgetary reserve needs, and offer care management services to high-risk individuals [1]. Preventing risk selection by insurers is a critical ethical, legal, and societal goal that risk adjustment models can address. Risk adjustment models attempt to capture the relationship between demographic and clinical variables (risk adjusters) and subsequent healthcare utilization or spending. The models are commonly derived through standard linear regression methods or their extensions, and rely on individual-level data commonly captured in administrative claims datasets [2]. All of the available models on the current commercial market are linear or log-linear regression models that leverage the same basic elements such as age, sex, diagnostic and procedure codes [3].

Risk adjustment modeling may be improved by both methodological and conceptual advances in the risk modeling and healthcare services literature. From a methodological standpoint, newer machine learning methods have recently emerged as alternatives or complements to linear regression for predicting highly variable health outcomes using large sparse datasets, including estimating healthcare costs using claims data [4, 5]. While traditional risk adjustment models are limited in modeling complexity and tend to underpredict expenditures of populations with very high expenditures [6, 7], machine learning methods may help to capture complex non-linear relationships and interaction terms among variables, which could explain why some individuals with complex constellations of risk factors and diagnoses experience substantially higher cost than predicted. For example, among people with low income and diabetes receiving insulin, food insecurity is associated with hypoglycemia and emergency room visits during the last week of each month (after income from a first-of-the-month paycheck is deprived) and hypoglycemic medications are still being taken [8]. These complex relationships are hard to model in standard risk equations, but can be potentially better captured by interactions-focused, nonlinear machine learning algorithms. Despite the promise of machine learning for risk adjustment, machine learning techniques have not yet been widely adopted for risk adjustment. This is partially because the machine learning models developed to date have not yet demonstrated superior predictive performance over traditional linear models on large datasets with more than a million enrollees [2].

From a conceptual standpoint, risk adjustment may also be improved by including additional area-level indicators of social determinants of health (SDH), such as poverty, unemployment, and education, which contribute to risk, utilization and cost [9–11]. Since before the UK Black Report and the Health Divide, epidemiologists have shown that while cultural and individual behavioral choices influence health, living conditions including the availability of resources (e.g., clean air and water), working conditions, and quality of food and housing have a particularly profound association with health outcomes [12]. More recent initiatives to directly address these ‘social determinants’ of health include strategies to refer patients with food insecurity to food pantries, those that are homeless to direct housing resources, and those with challenges with transportation to assisted transport services, as a means to improve clinical outcomes such as nutrition-related chronic disease metrics (e.g., nutrition affecting blood pressure and diabetes glycemic control) and to improve the ability to access healthcare visits and reduce stress-related adverse health outcomes [13].

The inclusion of SDH indicators into risk adjustment may particularly help plan payment estimation. SDH indicators may help capture previously unmeasured factors that could influence the course of disease, such as how poverty may affect chronic disease outcomes by affecting the ability to pay for medications or more nutritious foods, or how unemployment relates to mental health and associated course of disease related to depression and lower adherence [14, 15]. Individual-level SDH factors are rarely assessed or included in commonly-available data, but area-level SDH indicators are readily assessed by national data sources [16], and may be linked to the 5-digit ZIP code often available in claims data. Area-level SDH indicators were recently incorporated into risk adjustment models for the Massachusetts Medicaid program; their inclusion improved concurrent annual healthcare spending predictions for low-income adults [17]. It remains unclear, however, to what extent incorporating area-level SDH indicators could improve prospective annual healthcare spending predictions, particularly for the privately-insured population who constitute the largest share of insured people in the US, but for whom SDH factors may be less visible or influential than for the Medicaid population.

The objective of this study was to assess whether prospective risk adjustment models may be improved by machine learning methods and by the incorporation of area-level SDH indicators in a national privately-insured adult population.

## Method

### Data

**Fig. 1:**
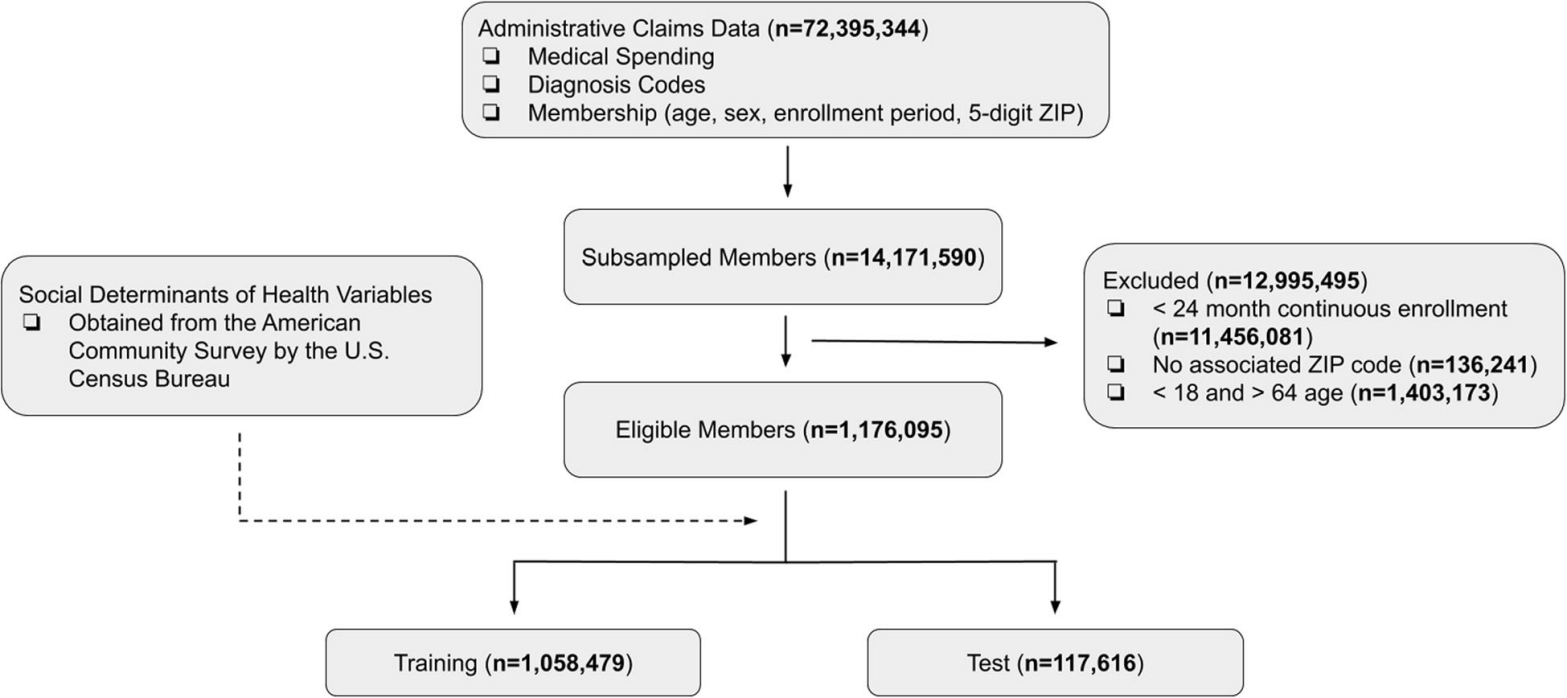
Data Set Selection Flow Diagram. Administrative claims data was obtained from a single national private insurer. The dotted arrow means predictors were optionally incorporated but no members were added or excluded

Our primary data were healthcare claims from a single large national commercial insurer operating in all 50 US states, Washington D.C., and Puerto Rico (Fig. 1). From the claims data, we included privately-insured individuals 18 through 64 years old who had at least 24 months of continuous enrollment. Individuals who switched plans were implicitly excluded due to the continuous enrollment criteria, but individuals who moved were included. We used demographics (age, sex) and diagnostic codes (Clinical Classification Software categories [18]) as candidate risk adjustment variables at the individual level, and SDH indicators at the 5-digit ZIP code-level from the American Community Survey (ACS) by the U.S. Census Bureau (Table 1) [16]. SDH indicators were selected to reflect current conceptual theories concerning a broad range of social, economic, and health system factors that may influence health risk, utilization, or cost (Supplementary Information Table S1) [19, 20]. The resulting dataset contained claims data from 1.18 million unique members, which we randomly partitioned into training data (1.06 million members) for model derivation and test data (0.12 million members) for model performance assessment [21]. There was no overlap of individuals among the two data subsets. Demographic statistics of the data subsets by geographic location are reported in Supplementary Information Table S2.

**Table 1:** Statistics of ZIP Code-Level Social Determinants of Health

SDH Variable	Mean [Median] (Std)
Median Income in the Past 12 Months, $	26,546 [25727] (6230)
Families Under 0.5 Ratio of Income to Poverty Level in the Past 12 Months, %	4.7 [4.3] (2.7)
Families Between 0.5 and 0.74 Ratio of Income to Poverty Level in the Past 12 Months, %	3.2 [3.0] (1.7)
Families Between 0.75 and 0.99 Ratio of Income to Poverty Level in the Past 12 Months, %	3.6 [3.4] (1.6)
Families Received Food Stamps/Snap in the Past 12 months, %	14.2 [13.6] (6.6)
Population Unemployed, %	5.4 [5.2] (1.9)
Gini Index of Income Inequality	45.2 [45.1] (3.6)
Population Obtained High School Diploma, %	43.0 [42.9] (4.8)
Population Obtained Bachelor’s Degree, %	16.1 [15.2] (6.2)
Population Speak English Less than “Very Well”, %	10.5 [5.6] (12.5)
Families with Single Parent, %	22.9 [22.7] (6.2)
Population Without Health Insurance Coverage, %	11.3 [10.6] (4.9)
Population African American, %	9.9 [4.5] (13.5)
Population Asian, %	2.9 [1.2] (4.7)
Population American Indian and Alaska Native, %	1.5 [0.3] (6.4)
Population Hispanic or Latino, %	11.9 [5.5] (15.8)
Population White, %	71.8 [77.7] (22.3)

SDH variables were obtained from the 2012–2016 American Community Survey 5-year estimates from the U.S. Census Bureau

### Outcome

We sought to prospectively predict 2017 individual-level total annual healthcare spending from 2016 data. As a secondary objective, we also considered concurrent risk adjustment, predicting 2016 member-level annual spending from 2016 data, as is done, for instance, in the Affordable Care Act health insurance exchanges (see Supplementary Information). We estimated total annual spending by summing standardized costs in U.S. Dollars over 12 months, including post-year claims corrections, and including zero spending among enrolled individuals without medical claims. To inhibit outliers from affecting model fit, costs in the training set were top-coded at $400,000 (cost larger than $400,000 was replaced with $400,000), which corresponded to the top 0.1% cost of members in the training set. Top-coding is performed to reduce model sensitivity to skewness and kurtosis, and has been preferred over dropping members with high cost since these cases can be indicative of specific conditions which are associated with high cost [2, 22, 23].

### Model development

A 2-by-2 factorial design was employed to compare modeling approaches (linear regression versus the machine learning approach of gradient boosted decision trees), and variable choice (demographics and diagnostic codes alone versus additional area-level SDH indicators). In each of the methods, individual-level predictors with their associated area-level predictors are input to the model together as if they were all individual-level properties.

#### Linear regression approach

A linear model derived through ordinary least squares regression was trained to predict 2017 spending based on 2016 member characteristics. We additionally developed penalized linear regression models using methods that may better address collinearity (Least Absolute Shrinkage and Selection Operator [LASSO]), as detailed in the Supplementary Information. LASSO regression tends to sparsely select among collinear variables by forcing coefficients to zero for all but one of the collinear variables [24, 25].

#### Machine learning approach

The machine learning approach investigated in this study was gradient boosted decision trees [26]. This approach involves the construction of an ensemble of decision trees, where each tree learns from the errors of the prior tree (a “boosting” approach) to iteratively improve predictions [27]. With each iteration, a new tree is constructed by sampling from the data and identifying which variable most effectively divides the members into groups with low within-group variation in cost and high between-group variation in cost. This variable selection process is repeated to further divide each resulting subset of the data, producing a series of branches in the decision tree. The tree is added to the current ensemble, and then the next tree is fit using the same process on the residuals of the ensemble.

We chose gradient boosted decision trees over alternative machine learning methods because the approach has been shown to handle mixes of categorical and continuous covariates, capture nonlinear relationships, and scale well to large amounts of data [28]. Moreover, it is straightforward to obtain variable importance rankings from the model, which may permit the approach to be more interpretable than many other machine learning methods, for which acceptability in a healthcare services context may critically depend on visualizing “black box” predictions [29]. We used the LightGBM framework to develop the models, which implements several algorithmic optimizations on standard gradient boosting to allow for additional training efficiency [30]. A detailed treatment of gradient boosted decision trees and LightGBM is provided in the Supplementary Information. We used 3-fold cross validation on the training data subset to select the parameters for the model, including the number of trees, the maximum depth of each tree, and the minimum level of loss reduction necessary to partition leaf nodes, based on which achieved the lowest mean squared error averaged across the 3 folds [21]. We then refitted the model to the full training set using the best parameters determined from 3-fold cross validation, which can further help reduce overfitting. We additionally developed random forest and shallow multilayer perceptron models using a similar training procedure, as detailed in the Supplementary Information [31, 32].

### Model testing and statistical analysis

We evaluated the performance of the prospective risk adjustment models on the test set. The performance metrics are detailed below.

#### Goodness of fit

We evaluated model goodness of fit using the coefficient of determination (R^2^) and the mean absolute error (MAE). We estimated the R^2^ with confidence intervals using the nonparametric bootstrap with 5000 bootstrap replicates [33], and the MAE with confidence intervals using a paired t-test.

#### Discrimination

We assessed discrimination using the concordance-statistic (C-statistic), a rank correlation metric for assessing the model’s ability to order members by their spending [34]. The C-statistic estimates the probability that, for a randomly selected pair of members, the member with the higher cost will be correctly predicted as having higher cost by the model [35]. The C-statistic is the generalization of the area under the receiver operating characteristic curve from the binary to the continuous outcome setting, where a result between 0.7 and 0.8 is considered acceptable, between 0.8 and 0.9 is considered good, and above 0.9 is considered excellent [36]. We estimated confidence intervals for the C-statistic using a jack-knife procedure [37].

#### Subgroup analyses

Risk adjustment models often underpredict spending for specific subgroups of enrollees leading to underpayment to the insurer, and there is evidence that insurers explicitly make health plans less desirable for enrollees in undercompensated groups [38, 39]. To evaluate the performance of the models on vulnerable subgroups, we defined test data subgroups using age, sex, and area-level SDH indicators. SDH indicator subgroups included individuals living in ZIP codes in the lowest decile of household income; lowest decile of education level (by high school diploma and by bachelor’s degree receipt); highest decile of Gini index for inequality; low ratio of income to poverty level; high proportion of households receiving food stamps; high proportion with single parents; high unemployment; high uninsurance rate; and high proportion reporting they do not speak English “very well” (see Supplementary Information Table S1 for decile thresholds).

The performance of the model on each subgroup was measured using the predictive ratio [40] and net compensation [39, 41]. The predictive ratio for a subgroup was computed as the ratio of the mean of observed spending to the mean of predicted spending over the subgroup, where a value above 1 indicates underestimation of cost and a value below 1 indicates overestimation [17]. We estimated 95% confidence intervals around the predictive ratio using the delta method [42]. Net compensation was used as a measure on the dollar scale, and was computed as the mean difference between predicted spending and observed spending over the subgroup, where a value below 0 indicates underestimation of cost and a value over 0 indicates overestimation. We estimated 95% confidence intervals around the net compensation values using a paired t-test.

Analyses were approved by the Stanford Institutional Review Board (eProtocol #42334), and performed in Python version 3.6.6 [43] and R version 3.5.0 [44], using the code shared online for reproducibility at: https://github.com/stanfordmlgroup/risk-adjustment-ml.

## Results

Descriptive statistics on the data subsets are detailed in Table 2. The test set had a mean age of 41.1 years (median 41.0; IQR 30.0, 53.0) and was 48.9% female. Top-coding cost at $400,000 eliminated approximately 2.8% of dollars and test set members had a mean top-coded annual healthcare cost of $6677 (median 855; IQR 161, 3847). Around 17.7% of members in the test set had zero annual healthcare cost.

**Table 2:** Characteristics of Members in the Dataset Subsets

Characteristic	Training Set	Test Set
Members Total, No.	1,058,479	117,616
Female Total, No. (%)	517,364 (48.9%)	57,469 (48.9%)
Members from ZIP codes without measured SDH variables^a^, No. (%)	1074 (0.1%)	115 (0.1%)
Population statistics, mean [median] (SD)		
Age, y	41.1 [41.0] (13.1)	41.1 [41.0] (13.1)
2017 Annual Cost, $	6946 [861] (28,240)	6868 [855] (27,826)
2017 Top-coded Annual Cost^b^, $	6762 [861] (23,822)	6677 [855] (23,536)

The training set was used to develop the models and the test set was used to evaluate the models

^a^The SDH variables of these members were imputed with the median values of SDH variables over all ZIP codes, and an additional indicator variable was used to identify whether members fall into this category

^b^Statistics of cost when top-coding at $400,000 (values higher than $400,000 were replaced with $400,000)

Table 3 shows the test set performance of the prospective linear and machine learning models without and with the SDH indicators.

**Table 3:** Performance Measures of the Prospective Linear and Machine Learning Models on the Test Set

Evaluation Metric	No SDH	SDH
R^2^ (95% CI)^a^		
Linear	0.327 (0.300, 0.353)	0.327 (0.300, 0.354)
ML	0.388 (0.357, 0.420)	0.387 (0.357, 0.419)
MAE (95% CI)^b^		
Linear	6992 (6889, 7094)	6991 (6889, 7094)
ML	6637 (6539, 6735)	6634 (6536, 6732)
C-statistic (95% CI)^c^		
Linear	0.703 (0.701, 0.705)	0.700 (0.699, 0.702)
ML	0.717 (0.715, 0.718)	0.716 (0.714, 0.717)

Comparison of performance measures between linear regression and machine learning prospective risk adjustment models, predicting 2017 yearly top-coded spending from 2016 characteristics. The SDH model additionally includes SDH variables obtained from U.S. Census data (see Table 1)

^a^Confidence intervals for R^2^ were constructed using the nonparametric bootstrap [21]

^b^Confidence intervals for MAE were constructed using a paired t-test

^c^Confidence intervals for C-statistic were constructed using a jackknife procedure [25]

### Linear regression without SDH indicators

The linear regression model without SDH indicators, when derived through ordinary least squares regression, had the largest standardized coefficients (indicating highest importance among covariates in the model) for age and sex indicators and diagnostic coding for birth complications and chronic kidney disease (see Supplementary Information Table S3). The model had a R^2^ of 0.327 (95% CI 0.300, 0.353), MAE of $6992 (95% CI 6889, 7094), and C-statistic of 0.703 (95% CI 0.701, 0.705). Linear models derived through LASSO had similar performance metrics but tended to favor diagnoses more than traditional least squares (see Supplementary Information Tables S3 and S5).

### Linear regression with SDH indicators

The inclusion of SDH indicators in the linear regression model had no substantial effect on the overall performance metrics. The model had a R^2^ 0.327 (95% CI 0.300, 0.354), MAE of $6991 (95% CI 6889, 7094), and C-statistic of 0.700 (95% CI 0.699, 0.702).

### Machine learning without SDH indicators

Switching from a linear regression model to the machine learning model significantly improved determination, significantly reduced error, and significantly improved discrimination. Specifically, the machine learning model without SDH indicators had a R^2^ of 0.388 (95% CI 0.357, 0.420), MAE of $6637 (95% CI 6539, 6735), and C-statistic of 0.717 (95% CI 0.715, 0.718). The multilayer perceptron and random forest models outperformed the linear models but performed worse than the LightGBM model across all metrics (Supplementary Information Table S5).

### Machine learning with SDH indicators

The inclusion of SDH indicators in the machine learning model also had no substantial effect on the overall performance metrics above the machine learning model without SDH indicators. The model had a R^2^ of 0.387 (95% CI 0.357, 0.419), MAE of $6634 (95% CI 6536, 6732), and C-statistic of 0.716 (95% CI 0.714, 0.717). We created variable importance rankings to assist in the interpretation of the machine learning model. Diagnosis predictors had the largest importance metrics in the machine learning model, with the most important predictors being chronic kidney disease, deficiency and other anemia, and other aftercare (see Supplementary Information Table S4).

### Subgroup analyses

Table 4 compares the predictive ratios and net compensation values for the machine learning model without and with SDH indicators. The addition of SDH indicators resolved or reduced underestimation of risk on all of the SDH-based subgroups, but the 95% confidence intervals were overlapping between the non-SDH and SDH-including models among all subgroups. On one of the high-poverty subgroups, the subgroup with a high proportion of non-fluent English speakers, the subgroup with a high prevalence of uninsured, and the subgroup of individuals who lived in areas with a large proportion of households on food stamps, the incorporation of SDH indicators resolved the underestimation of risk. Among subgroups of individuals who lived in areas with high poverty, high wealth inequality, and high prevalence of uninsured, the machine learning model trained with SDH indicators substantially reduced underestimation of cost among the subgroup, improving the predictive ratio by 3% (and net compensation by $200 per person) over the model trained without SDH indicators. The addition of SDH indicators led to small additional overpayment on the 4 subgroups for which the model without SDH indicators did not substantially underestimate risk (predictive ratio < 1.01), specifically one of the high-poverty subgroups, the subgroup with a large unemployed population, the subgroup with a low percentage of high school graduates, and the subgroup with a large number of single-parent families. Additional subgroup analyses among all models are presented in Supplementary Information Tables S6, 7, 8.

**Table 4:** Predictive Ratio and Net Compensation Values of Prospective Machine Learning Models on SDH-Based Subgroups in the Test Set

			Model Predictive Ratio^b^ and Net Compensation^c^	
Subgroup	No. (%)	2017 Spending ($)^a^	ML (95% CI)	ML with SDH (95% CI)
Total	117,616 (100)	6677	1.000 (0.976, 1.024)	1.000 (0.976, 1.024)
			0 (− 105, 105)	0 (− 105, 105)
Poverty				
Median Income in the Past 12 Months, $	4923 (4.2)	10,818	1.017 (0.915, 1.120)	1.006 (0.905, 1.108)
			− 183 (− 836, 470)	−67 (− 729, 595)
Families Under 0.5 Ratio of Income to Poverty Level in the Past 12 Months, %	7932 (6.7)	9344	0.966 (0.882, 1.050)	0.948 (0.865, 1.031)
			331 (− 138, 801)	510 (33, 987)
Families Between 0.5 and 0.74 Ratio of Income to Poverty Level in the Past 12 Months, %	6651 (5.7)	8952	1.010 (0.912, 1.108)	0.988 (0.892, 1.084)
			−89 (− 599, 420)	109 (− 408, 627)
Families Between 0.75 and 0.99 Ratio of Income to Poverty Level in the Past 12 Months, %	7194 (6.1)	9395	1.052 (0.956, 1.148)	1.010 (0.919, 1.101)
			− 467 (− 977, 43)	−94 (− 613, 425)
Families Received Food Stamps/Snap in the Past 12 months, %	9009 (7.7)	9001	1.028 (0.941, 1.115)	0.996 (0.912, 1.079)
			− 247 (− 684, 191)	39 (− 409, 487)
Population Unemployed, %	10,278 (8.7)	7055	0.961 (0.886, 1.036)	0.957 (0.882, 1.032)
			289 (−71, 649)	316 (−51, 683)
Gini Index of Income Inequality	16,155 (13.7)	6138	1.054 (0.985, 1.122)	1.021 (0.955, 1.087)
			− 312 (− 578, −46)	− 126 (− 393, 140)
Education				
Population Obtained High School Diploma, %	9482 (8.1)	7555	0.987 (0.900, 1.073)	0.974 (0.889, 1.058)
			102 (− 324, 529)	205 (− 227, 637)
Population Obtained Bachelor’s Degree, %	4169 (3.5)	11,338	1.032 (0.923, 1.142)	1.027 (0.917, 1.136)
			−353 (− 1139, 433)	− 294 (− 1080, 492)
Other				
Population Speak English Less than “Very Well”, %	23,659 (20.1)	5453	1.023 (0.963, 1.083)	0.989 (0.932, 1.046)
			− 124 (− 346, 98)	61 (−161, 283)
Families with Single Parent, %	9097 (7.7)	9880	0.993 (0.910, 1.076)	0.978 (0.896, 1.060)
			65 (− 397, 527)	224 (− 246, 693)
Population Without Health Insurance Coverage, %	13,656 (11.6)	8333	1.066 (0.990, 1.142)	0.990 (0.921, 1.059)
			− 516 (− 885, − 147)	83 (− 287, 454)

Comparison of machine learning prospective risk adjustment models without and with the addition of SDH indicators as predictors (see Table 1 for a complete list of SDH indicators). The predictions for each model were adjusted so that the mean of the predictions over the total test population was equal to the mean of the actual costs, resulting in a predictive ratio of exactly 1.0 over the total test set population. Subgroups were composed of members in the lowest decile of ZIP codes with respect to the corresponding SDH variable (see Supplementary Information Table S1). Only socioeconomic variables are considered in this subgroup analysis, and results on age and sex subgroups are shown in the Supplementary Information

^a^Spending included all healthcare utilization in 2017 of members with full enrollment in 2016 and 2017. Values larger than $400,000 were replaced with $400,000

^b^Predictive ratio for a subgroup was computed as the ratio of the mean of observed to the mean of predicted spending over the subgroup. Approximate confidence intervals for predictive ratios were computed with the delta method [40]

^c^Net compensation for a subgroup was computed as the mean difference between predicted and observed spending in the subgroup. Confidence intervals were estimated using a paired t-test

### Additional results

Binned scatter plots of the prospective risk adjustment models on the test set are shown in Fig. S1. We additionally explored the effect of using binary diagnosis predictors instead of counts (Supplementary Information Table S9), the effect of top-coding cost (Supplementary Information Table S10), the effect of including lab results (Supplementary Information Table S11), and the development of concurrent risk adjustment models (Supplementary Information Table S12).

## Discussion

We observed that switching from a linear regression model to a gradient boosting ML model significantly improved determination and discrimination and reduced absolute error in cost. We also observed that the inclusion of SDH indicators at the ZIP code-level reduced underestimation of cost among people living in vulnerable areas.

Prior studies have separately investigated whether machine learning and the incorporation of SDH indicators can improve risk adjustment. The use of machine learning for prospective risk prediction in a previous study did not demonstrate substantial improvements over linear regression for a privately-insured population [4]. However, the addition of SDH indicators has been shown to improve concurrent risk adjustment models, including Medicare Advantage Plan quality rankings, Medicare’s Hospital Readmissions Reduction Program penalties, and concurrent annual healthcare spending among a state Medicaid population [17, 45, 46]. In our study, the incorporation of SDH indicators reduced cost underestimation in several vulnerable subgroups, even among a commercially-insured population. Improving predictions of cost within these subgroups is important in order to address persistent inequalities that lead to bias in the estimation of payment [47–49].

Our study has important limitations. First, the risk models developed here are unlikely to generalize well to populations outside the U.S. as well as to Medicaid or Medicare populations for whom risk adjustment models may be particularly consequential to avoid adverse selection and maintain competitive and fair markets. However, the methods employed in this study could be used in developing specific models for those populations. Second, similar to other machine learning methods, the modeling approach used in this study is more complex than traditional linear regression. Although this may confer an advantage due to the potential of preventing ‘cheating’, in that machine learning models may be less susceptible to up-coding behaviors intended to inflate risk estimates [2], the complexity might also contribute to difficulty to understand how and why the model made a certain decision [29]. Third, since risk adjustment models are developed on historical data, they tend to perpetuate inequality of past spending trends if no explicit adjustments are made to account for the endogeneity of spending. Prior work has investigated methods to develop fairer healthcare payment models through data manipulation and modeling changes [39, 41, 50], which can be pursued in future studies. Fourth, the SDH indicators used in this study are at the area-level which may lead to bias or ecological fallacy in the risk adjustment models. However, combining the claims data used in this work with individual-level socioeconomic status variables was prohibited for privacy reasons. Fifth, 5-digit ZIP codes are not as homogeneous as Census Tracts or Census Block Groups, which have been used in previous linear regression models assessing SDH-associated effects for Medicaid and Medicare populations [51]. The risk for this study is a potential underestimation of the contribution of SDH to risk models. However, ZIP code is more readily available in commercial claims datasets. Sixth, there remains debate about whether adding in SDH indicators may allow for poorer healthcare to persist in healthcare organizations serving predominantly lower-income populations, by compensating them more in value-based payment models that adjust not only for outcomes but also for lower income for instance, although recent studies suggest this will not necessarily mask hospital quality [52]. Seventh, one key challenge is to predict per-member utilization rather than cost. However, given that cost is a key concern for payers and often disproportionate to utilization due to negotiated contracts and geographic variations in cost, we modeled overall costs to help understand how much geographic parameters such as social determinants and machine learning could capture the complexities related to payment.

In the future, our ML approach may be improved upon in several ways. It may be possible to take advantage of the temporality of the data, for example by including more than one year of medical history. Additionally, it may be possible to train a hybrid (concurrent and prospective) model to leverage the continuous nature of medical enrollment, utilization, and claims [53]. Finally, using highly parameterized models such as deep neural networks could better capture nonlinear interactions between covariates and scale to large claims datasets, at the expense of interpretability [54]. We have shared our code in an open source manner to enable others to reproduce and extend our methods to other datasets.

## Conclusion

The results of the current study suggest that machine learning methods and the inclusion of area-level SDH indicators may improve prospective risk adjustment models in a commercially insured population. The SDH indicators were particularly useful for populations living in vulnerable areas, while the machine learning approach had a greater impact on overall performance, leading to improvements in fit, discrimination, and overall cost allocation (>$3 M reduction in error per 10,000 people).

## Supplementary information

**Additional file 1:** Appendix.  Additional details on the administrative claims dataset, input predictors, machine learning models, linear regression models, and statistical analysis. **Table S1**. Definitions and Conceptual Justification of the SDH Indicators. **Table S2** Demographic Statistics of the Data Subsets by Geographic Location **Table S3.** Variable Importances of the Prospective Linear Regression, LASSO Regression, Random Forest, and LightGBM Models without SDH Indicators **Table S4.** Variable Importances of the Prospective Linear Regression, LASSO Regression, Random Forest, and LightGBM Models with SDH Indicators **Table S5.** Performance Measures of LASSO Regression, Random Forest, and Multilayer Perceptron on the Test Set **Table S6.** Predictive Ratio and Net Compensation Values of Prospective Machine Learning Models on Age and Sex Subgroups in the Test Set **Table S7.** Predictive Ratio and Net Compensation Values of Prospective Linear Models on SDH-Based Subgroups in the Test Set **Table S8.** Predictive Ratio and Net Compensation Values of Prospective Linear Models on Age and Sex Subgroups in the Test Set **Figure S1.** Binned Scatter Plots of the Prospective Linear Regression and Machine Learning Models without and with SDH Indicators on the Test Set **Table S9.** Performance Measures of Models Derived Using Binary Diagnosis Predictors on the Test Set **Table S10.** Performance Measures of Top-Coded and Non-Top-Coded Models on the Test Set **Table S11.** Performance Measures of Models with Lab Results on the Test Set **Table S12.** Performance Measures of Concurrent and Prospective Models with SDH Indicators on the Test Set

## Data Availability

The code used in this study is shared online for reproducibility at: https://github.com/stanfordmlgroup/risk-adjustment-ml .

## Abbreviations

ML: Machine learning
SDH: Social determinants of health
ACS: American Community Survey
MAE: Mean absolute error
C-statistic: Concordance statistic
CI: Confidence interval
