# Supplementary material for: Incorporating machine learning and social determinants of health indicators into prospective risk adjustment for health plan payments
Source: BMC Public Health. 2020 May 1;20:608. doi: 10.1186/s12889-020-08735-0 (PMC7195714; doi:10.1186/s12889-020-08735-0)
Supplement: Supplementary file 1 — Additional file 1: Appendix. Additional details on the administrative claims dataset, input predictors, machine learning models, linear regression models, and statistical analysis. Table S1. Definitions and Conceptual Justification of the SDH Indicators. Table S2 Demographic Statistics of the Data Subsets by Geographic Location Table S3. Variable Importances of the Prospective Linear Regression, LASSO Regression, Random Forest, and LightGBM Models without SDH Indicators Table S4. Variable Importances of the Prospective Linear Regression, LASSO Regression, Random Forest, and LightGBM Models with SDH Indicators Table S5. Performance Measures of LASSO Regression, Random Forest, and Multilayer Perceptron on the Test Set Table S6. Predictive Ratio and Net Compensation Values of Prospective Machine Learning Models on Age and Sex Subgroups in the Test Set Table S7. Predictive Ratio and Net Compensation Values of Prospective Linear Models on SDH-Based Subgroups in the Test Set Table S8. Predictive Ratio and Net Compensation Values of Prospective Linear Models on Age and Sex Subgroups in the Test Set Figure S1. Binned Scatter Plots of the Prospective Linear Regression and Machine Learning Models without and with SDH Indicators on the Test Set Table S9. Performance Measures of Models Derived Using Binary Diagnosis Predictors on the Test Set Table S10. Performance Measures of Top-Coded and Non-Top-Coded Models on the Test Set Table S11. Performance Measures of Models with Lab Results on the Test Set Table S12. Performance Measures of Concurrent and Prospective Models with SDH Indicators on the Test Set [file 12889_2020_8735_MOESM1_ESM.docx]

**Supplementary Information**

**Appendix.**

Administrative Claims Dataset

Predictors

Age and Sex Predictors

Diagnosis Predictors

Social Determinants of Health Predictors

Lab Results Predictors

Machine Learning Details

Gradient Boosting

LightGBM

Penalized Linear Regression

Statistical Analysis Implementation

Additional Results

**References.**

**Table S1.** Definitions and Conceptual Justification of the SDH Indicators

**Table S2.** Demographic Statistics of the Data Subsets by Geographic Location

**Table S3.** Variable Importances of the Prospective Linear Regression, LASSO Regression, Random Forest, and LightGBM Models without SDH Indicators

**Table S4.** Variable Importances of the Prospective Linear Regression, LASSO Regression, Random Forest, and LightGBM Models with SDH Indicators

**Table S5.** Performance Measures of LASSO Regression, Random Forest, and Multilayer Perceptron on the Test Set

**Table S6.** Predictive Ratio and Net Compensation Values of Prospective Machine Learning Models on Age and Sex Subgroups in the Test Set

**Table S7.** Predictive Ratio and Net Compensation Values of Prospective Linear Models on SDH-Based Subgroups in the Test Set

**Table S8.** Predictive Ratio and Net Compensation Values of Prospective Linear Models on Age and Sex Subgroups in the Test Set

**Figure S1.** Binned Scatter Plots of the Prospective Linear Regression and Machine Learning Models without and with SDH Indicators on the Test Set

**Table S9.** Performance Measures of Models Derived Using Binary Diagnosis Predictors on the Test Set

**Table S10.** Performance Measures of Top-Coded and Non-Top-Coded Models on the Test Set

**Table S11.** Performance Measures of Models with Lab Results on the Test Set

**Table S12.** Performance Measures of Concurrent and Prospective Models with SDH Indicators on the Test Set

**Appendix**

**Administrative Claims Dataset**

A healthcare claims dataset from a large national commercial insurer was used to develop and validate the risk adjustment models. The insurer operates in all 50 states, Washington D.C., and Puerto Rico and members from each geographic location are represented in both the training and test sets used in this work. Demographic statistics of the members across each of the locations are shown in **Table S2**.

**Predictors**

We included 12 age and sex predictors and 205 diagnosis predictors constituting 217 predictors in the models without SDH indicators, and optionally included 18 additional SDH indicator variables constituting 235 predictors in the models with SDH indicators. Predictors are described in detail below.

**Age and Sex Predictors**

We incorporated several age and sex indicators as predictors in the risk adjustment models. To capture nonlinear interactions between age and sex covariates, we included interaction terms similar to those used in the risk adjustment model recently implemented by the Massachusetts Medicaid program (see **Table S6**).^1^ We used 6 age buckets for each sex, constituting 12 age-sex interaction variables in total.

**Diagnosis Predictors**

We included diagnostic codes from 2016 as predictors in the risk adjustment models. Diagnoses were coded in the claims dataset using the 10th revision of International Classification of Diseases, Clinical Modification (ICD-10-CM). We categorized the ICD-10-CM diagnosis codes into Clinical Classification Software (CCS) codes created by the Agency for HealthCare Quality and Research^2^. We excluded 79 CCS categories which occurred in less than 1% of all members in the training set:

Tuberculosis; Cancer of esophagus; Cancer of stomach; Cancer of liver and intrahepatic bile duct; Cancer of pancreas; Cancer of other GI organs; peritoneum; Cancer; other respiratory and intrathoracic; Cancer of bone and connective tissue; Cancer of uterus; Cancer of ovary; Cancer of other female genital organs; Cancer of testis; Cancer of other male genital organs; Cancer of bladder; Cancer of kidney and renal pelvis; Cancer of other urinary organs; Cancer of brain and nervous system; Hodgkin`s disease; Cancer; other and unspecified primary; Malignant neoplasm without specification of site; Cystic fibrosis; Meningitis (except that caused by tuberculosis or sexually transmitted disease); Encephalitis (except that caused by tuberculosis or sexually transmitted disease); Other CNS infection and poliomyelitis; Parkinson`s disease; Coma; stupor; and brain damage; Cardiac arrest and ventricular fibrillation; Aortic and peripheral arterial embolism or thrombosis; Aspiration pneumonitis; food/vomitus; Lung disease due to external agents; Peritonitis and intestinal abscess; Nephritis; nephrosis; renal sclerosis; Spontaneous abortion; Induced abortion; Postabortion complications; Ectopic pregnancy; Early or threatened labor; Prolonged pregnancy; Malposition; malpresentation; Fetopelvic disproportion; obstruction; Fetal distress and abnormal forces of labor; Umbilical cord complication; Forceps delivery; Pathological fracture; Digestive congenital anomalies; Genitourinary congenital anomalies; Nervous system congenital anomalies; Liveborn; Short gestation; low birth weight; and fetal growth retardation; Intrauterine hypoxia and birth asphyxia; Respiratory distress syndrome; Hemolytic jaundice and perinatal jaundice; Birth trauma; Other perinatal conditions; Fracture of neck of femur (hip); Spinal cord injury; Skull and face fractures; Crushing injury or internal injury; Poisoning by psychotropic agents; Poisoning by other medications and drugs; Poisoning by nonmedicinal substances; Gangrene; Shock; Impulse control disorders NEC; External cause codes: Cut/pierce; External cause codes: Drowning/submersion; External cause codes: Fire/burn; External cause codes: Firearm; External cause codes: Machinery; External cause codes: Pedal cyclist; not MVT; External cause codes: Pedestrian; not MVT; External cause codes: Transport; not MVT; External cause codes: Overexertion; External cause codes: Poisoning; External cause codes: Struck by; against; External cause codes: Suffocation; Adverse effects of medical care; External cause codes: Other specified and classifiable; External cause codes: Other specified; NEC

In total we incorporated 204 CCS codes as covariates, and if an ICD-10 code is not categorized into any CCS category, it was categorized as “Other.” We aggregated diagnoses over the year for each member by maintaining counts of all CCS categories coded (and “Other”) in that year (a “Bag-of-Words” representation^3^). In **Table S9**, we additionally investigate the effect of encoding CCS predictors as binary indicators instead of counts (a “boolean Bag-of-Words” representation).

**Social Determinants of Health Predictors**

We used the 5-digit ZIP code of the member enrollment period to merge SDH covariates from American Census Survey (ACS) by the U.S. Census Bureau.^4^ If a patient is listed as having multiple ZIP codes in an enrollment period, we selected the first available ZIP code. We incorporated 17 SDH variables (Table 1), each described in detail in the ACS Data Dictionary. All SDH predictors are continuous variables representing percentages, except for the median income predictor which is represented in dollars.

Since survey estimates are available at the GEOID-level, we converted GEOID to ZCTA using a mapping from the U.S. Census Bureau, and then ZCTA to ZIP code using a mapping from the UDS Mapper.^5^ Since each GEOID may have multiple ZCTA, we one-to-one match GEOID to ZCTA using closest haversine distance based on longitude and latitude of GEOID and ZCTA population centroids. For any variables that correspond to counts of population, families, or households, we computed percentages by normalizing by the total of the associated “universe”. For members from ZIP codes without measured SDH variables, the SDH variables were imputed with the median values of SDH variables over all ZIP codes, and an additional indicator variable was used to identify whether members fall into this category.

**Lab Results Predictors**

We included lab results data in the prospective risk adjustment models in a sensitivity analysis. We used the type of lab test ordered and whether the test was normal, high, low, or abnormal if no specific information about the level of abnormality was provided. We considered the top 500 most common test type and result pairs as well as “Normal Unknown Lab Result”, and any pair which is not in the top 500 or coded as this unknown category was categorized as “Other.” We aggregated lab results over the year for each member by maintaining counts of all lab tests and results which were obtained (and “Other”) in that year.

**Machine Learning Details**

In this study, we used gradient boosted decision trees, a class of machine learning models that iteratively build an ensemble of weak predictors to construct a model better than any individual predictor (a “boosting” approach). This machine learning method is especially effective due to its ability to handle mixes of categorical and continuous covariates, capture nonlinear relationships, and scale well to large amounts of data.^6^

The particular type of weak predictor used in this study was a decision tree.^7^ In a basic decision tree model, branches are iteratively constructed by sampling from the data and first identifying which variable most effectively divides the members into groups with low within-group variation in the outcome (cost in this study) and high between-group variation in outcome. Then, the variable selection process is repeated to further divide each resulting subset of the data recursively, producing a series of branches in the decision tree. In the regression setting, the mean value of the sample data is used as the final prediction per region.

**Gradient Boosting**

To combine independent weak learners like decision trees, a standard approach is to use an unweighted linear combination of their predictions (an ensemble^8^), which is the “Super Learner” approach used in Rose et al.^9^ Gradient boosting, however, automatically learns to grow and iteratively add on new decision trees to constitute the ensemble model, whose predictions are a weighted combination of the predictions from each decision tree. Formally, gradient boosting is a coordinate descent method that incentivizes each subsequent learner to improve performance on underperforming subsets of the training data measured by a specified loss function *L*. Given the current ensemble of learners $f_{j-1}$ and it’s prediction on input $x$, $f_{j-1}\left( x \right)$, we define the next iteration of the ensemble $f_{j}\left( x \right)$ as the current ensemble added to the learner $h_{j}$ that best minimizes the residual error $y-f_{j-1}\left( x \right)$:

$$f_{j}\left( x \right)=f_{j-1}\left( x \right)+\text{argmin}_{h_{j}\in\mathcal{H}}\left[ \sum_{i=1}^{n} L\left( y_{i},f_{j-1}\left( x_{i} \right)+h_{j}\left( x_{i} \right) \right) \right]$$

The loss is minimized by training each subsequent decision tree to predict the residuals of the loss with respect to the cumulative prediction of the previous learners. This procedure can be interpreted as minimizing the loss by taking gradients in function space.^10^ In this study, the loss function was the mean squared error between the predicted and observed costs. Intuitively, each subsequent learner can be interpreted as attempting to accommodate for the mistakes of the previous one by adding a “nudge” to each prediction along the direction that minimizes the loss. A coefficient for each weak learner is also learned automatically, and more sophisticated gradient boosting algorithms learn coefficients per region of the learner, rather than a single coefficient per learner.

**LightGBM**

LightGBM is a framework that enables GPU-accelerated learning of gradient boosted decision trees.^11^ The primary difference of LightGBM from other gradient boosting frameworks is that it grows decision trees “leaf-wise”, such that subsequent learners are not always minimizing the pseudo-residuals of the previously added learner. This has been shown to improve model performance with a larger risk of overfitting, which was unlikely in our large data setting. LightGBM additionally includes GPU acceleration for decision trees, implementing a highly parallelized tree building procedure.^6^ We trained each model on a single NVIDIA Titan Xp GPU, and each took around 30 seconds to fit the training set and 5 seconds to run on the test set.

**Penalized Linear Regression**

We developed penalized linear regression models using LASSO in addition to the ordinary least squares models. For each of these models, we used 3-fold cross validation on the training set to choose the regularization coefficient for LASSO. The parameters were chosen based on which achieved the lowest mean squared error averaged across the 3 folds. We then refitted each of the models using the best parameters on the full training set. Compared to traditional linear regression which took 1 minute to fit each model on the training set, LASSO regression took 12.4 minutes on average to fit. Both models took less than 2 seconds to run on the test set.

**Random Forest and Multilayer Perceptron**

We additionally developed random forest and multilayer perceptron models.^12,13^ For the random forest models, we used 3-fold cross validation on the training set to select the number of estimators, number of features for determining the best split, the maximum tree depth, the minimum number of samples per split, the minimum number of samples per leaf, and whether to bootstrap the dataset when constructing trees. For the multilayer perceptron models, we used 3-fold cross validation on the training set to choose the number of hidden layers, hidden layer sizes, initial learning rate, and activation function. We experimented with multilayer perceptron models developed using all predictors and predictors selected by LASSO. The parameters for each model were chosen based on which achieved the lowest mean squared error averaged across the 3 folds. We then refitted each of the models using the best parameters on the full training set. Random forest models took 20 minutes on average to fit and multilayer perceptron 10 minutes. Both models took 5 seconds to run on the test set.

**Statistical Analysis Implementation**

All statistical analyses were performed in the R environment for statistical computing.^14^ The Hmisc package was used to compute C-statistic with confidence intervals,^15^ and the stats package was used to compute MAE and net compensation with confidence intervals. The nonparametric bootstrap and the delta method were implemented in Python to compute respectively the R^2^ values and predictive ratio values with confidence intervals.

**Additional Results**

Binned scatter plots of the prospective risk adjustment models on the test set are shown in Figure S1. We additionally report results using binary diagnosis predictors instead of counts (**Table S9**), not performing top-coding (**Table S10**), including lab results as additional predictors (**Table S11**) and concurrent frame models (**Table S12**).

**References**

1. Ash AS, Mick EO, Ellis RP, Kiefe CI, Allison JJ, Clark MA. Social Determinants of Health in Managed Care Payment Formulas. *JAMA Intern Med*. 2017;177(10):1424-1430. doi:10.1001/jamainternmed.2017.3317

2. Clinical Classifications Software (CCS) for ICD-10-PCS (beta version). https://www.hcup-us.ahrq.gov/toolssoftware/ccs10/ccs10.jsp. Accessed September 22, 2019.

3. Min X, Yu B, Wang F. Predictive Modeling of the Hospital Readmission Risk from Patients’ Claims Data Using Machine Learning: A Case Study on COPD. *Sci Rep*. 2019;9. doi:10.1038/s41598-019-39071-y

4. 2012-2016 American Community Survey 5-year estimates. U.S. Census Bureau. https://www.socialexplorer.com/data/ACS2016_5yr/metadata/?ds=ACS16_5yr. Published 2016. Accessed March 11, 2019.

5. ZCTA to ZIPCode Crosswalk - UDS Mapper. https://www.udsmapper.org/zcta-crosswalk.cfm. Accessed May 14, 2019.

6. Zhang H, Si S, Hsieh C-J. GPU Acceleration for Large-scale Tree Boosting. In: ; 2018.

7. Quinlan JR. Induction of Decision Trees. *Mach Learn*. 1986;1(1):81–106. doi:10.1023/A:1022643204877

8. Dietterich TG. Ensemble Methods in Machine Learning. In: *Multiple Classifier Systems*. Lecture Notes in Computer Science. Springer Berlin Heidelberg; 2000:1-15.

9. Rose S. A Machine Learning Framework for Plan Payment Risk Adjustment. *Health Serv Res*. 2016;51(6):2358-2374. doi:10.1111/1475-6773.12464

10. Mason SLR, Baxter J, Bartlett P, Frean M. Boosting Algorithms as Gradient Descent in Function Space. In: ; 1999.

11. Ke G, Meng Q, Finley T, et al. LightGBM: A Highly Efficient Gradient Boosting Decision Tree. In: Guyon I, Luxburg UV, Bengio S, et al., eds. *Advances in Neural Information Processing Systems 30*. Curran Associates, Inc.; 2017:3146–3154. http://papers.nips.cc/paper/6907-lightgbm-a-highly-efficient-gradient-boosting-decision-tree.pdf. Accessed March 11, 2019.

12. Breiman L. Random Forests. *Mach Lang*. 2001;45(1):5–32. doi:10.1023/A:1010933404324

13. Hinton GE. Connectionist learning procedures. *Artif Intell*. 1989;40(1):185-234. doi:10.1016/0004-3702(89)90049-0

14. R Core Team. R: A Language and Environment for Statistical Computing. 2019. https://www.R-project.org/. Accessed February 11, 2019.

15. Harrell FE, others with contributions from CD and many. *Hmisc: Harrell Miscellaneous*.; 2019. https://CRAN.R-project.org/package=Hmisc. Accessed April 9, 2019.

16. Project WHC. *Social Determinants of Health: The Solid Facts*. World Health Organization; 2003.

17. Stuckler D, Basu S, Suhrcke M, Coutts A, McKee M. Effects of the 2008 recession on health: a first look at European data. *The Lancet*. 2011;378(9786):124-125. doi:10.1016/S0140-6736(11)61079-9

18. Marmot M. Income inequality, social environment, and inequalities in health. *J Policy Anal Manage*. 2001;20(1):156-159. doi:10.1002/1520-6688(200124)20:1<156::AID-PAM2009>3.0.CO;2-9

19. Organización Mundial de la Salud. *Social Determinants of Health and Well-Being among Young People: Health Behaviour in School-Aged Children (HBSC) Study : International Report from the 2009-2010 Survey*. Copenhagen: World Health Organization, Regional Office for Europe; 2012.

20. Council NR, Education D of B and SS and, Education C on B and SS and, Population C on NS and C on. *Statistics on U.S. Immigration: An Assessment of Data Needs for Future Research*. National Academies Press; 1996.

21. Basu S, Rehkopf DH, Siddiqi A, Glymour MM, Kawachi I. Health Behaviors, Mental Health, and Health Care Utilization Among Single Mothers After Welfare Reforms in the 1990s. *Am J Epidemiol*. 2016;183(6):531-538. doi:10.1093/aje/kwv249

22. Card D, Dobkin C, Maestas N. The Impact of Nearly Universal Insurance Coverage on Health Care Utilization: Evidence from Medicare. *Am Econ Rev*. 2008;98(5):2242-2258. doi:10.1257/aer.98.5.2242

23. Nickens HW. The health status of minority populations in the United States. *West J Med*. 1991;155(1):27-32.

24. Ash AS, Ellis RP, Pope GC, et al. Using diagnoses to describe populations and predict costs. *Health Care Financ Rev*. 2000;21(3):7-28.

25. Tibshirani R, Efron B. *An Introduction to the Bootstrap*. CRC Press; 1994. http://citeseerx.ist.psu.edu/viewdoc/summary?doi=10.1.1.473.2742. Accessed February 22, 2018.

26. Newson R. Confidence Intervals for Rank Statistics: Somers’ D and Extensions. *Stata J*. 2006;6(3):309-334. doi:10.1177/1536867X0600600302

**Table S1. Definitions and Conceptual Justification of the SDH Indicators**

| SDH Variable | Conceptual Justification | Lowest Decile Threshold |
| --- | --- | --- |
| [Median Income in the Past 12 Months](https://www.socialexplorer.com/data/ACS2016_5yr/metadata/?ds=ACS16_5yr&table=B06011), $ | Poverty and Health Outcomes^16^ | 20182 |
| [Families Under 0.5 Ratio of Income to Poverty Level in the Past 12 Months](https://www.socialexplorer.com/data/ACS2016_5yr/metadata/?ds=ACS16_5yr&table=B17026), % | Poverty and Health Outcomes^16^ | 7.4 |
| [Families Between 0.5 and 0.74 Ratio of Income to Poverty Level in the Past 12 Months](https://www.socialexplorer.com/data/ACS2016_5yr/metadata/?ds=ACS16_5yr&table=B17026), % | Poverty and Health Outcomes^16^ | 5.2 |
| [Families Between 0.75 and 0.99 Ratio of Income to Poverty Level in the Past 12 Months](https://www.socialexplorer.com/data/ACS2016_5yr/metadata/?ds=ACS16_5yr&table=B17026), % | Poverty and Health Outcomes^16^ | 5.7 |
| [Families Received Food Stamps/Snap in the Past 12 months](https://www.socialexplorer.com/data/ACS2016_5yr/metadata/?ds=ACS16_5yr&table=B22007), % | Poverty and Health Outcomes^16^ | 22.4 |
| [Population Unemployed](https://www.socialexplorer.com/data/ACS2016_5yr/metadata/?ds=ACS16_5yr&table=C18120), % | Unemployment and Health Outcomes^17^ | 7.4 |
| [Gini Index of Income Inequality](https://www.socialexplorer.com/data/ACS2016_5yr/metadata/?ds=ACS16_5yr&table=B19083) | Income Inequality and Health Outcomes^18^ | 50.0 |
| [Population Obtained High School Diploma](https://www.socialexplorer.com/data/ACS2016_5yr/metadata/?ds=ACS16_5yr&table=B15003), % | Education and Health Outcomes^19^ | 37.1 |
| [Population Obtained Bachelor's Degree](https://www.socialexplorer.com/data/ACS2016_5yr/metadata/?ds=ACS16_5yr&table=B15003), % | Education and Health Outcomes^19^ | 8.8 |
| [Population Speak English Less than "Very Well",](https://www.socialexplorer.com/data/ACS2016_5yr/metadata/?ds=ACS16_5yr&table=B06007) % | Assimilation and Health Outcomes^20^ | 28.6 |
| [Families with Single Parent](https://www.socialexplorer.com/data/ACS2016_5yr/metadata/?ds=ACS16_5yr&table=B05009), % | Single Parenting and Health Outcomes^21^ | 29.8 |
| [Population Without Health Insurance Coverage](https://www.socialexplorer.com/data/ACS2016_5yr/metadata/?ds=ACS16_5yr&table=B27001), % | Insurance Cover and Health^22^ | 17.1 |
| [Population African American](https://www.socialexplorer.com/data/ACS2016_5yr/metadata/?ds=ACS16_5yr&table=B01001B), % | Minority Status and Health^23^ | N/A |
| [Population Asian](https://www.socialexplorer.com/data/ACS2016_5yr/metadata/?ds=ACS16_5yr&table=B01001D), % | Minority Status and Health^23^ | N/A |
| [Population American Indian and Alaska Native](https://www.socialexplorer.com/data/ACS2016_5yr/metadata/?ds=ACS16_5yr&table=B01001C), % | Minority Status and Health^23^ | N/A |
| [Population Hispanic or Latino](https://www.socialexplorer.com/data/ACS2016_5yr/metadata/?ds=ACS16_5yr&table=B01001I), % | Minority Status and Health^23^ | N/A |
| [Population White](https://www.socialexplorer.com/data/ACS2016_5yr/metadata/?ds=ACS16_5yr&table=B01001H), % | Minority Status and Health^23^ | N/A |

Abbreviations: SDH, Social Determinants of Health

Definitions through American Community Survey links and conceptual justification of each of the ZIP code-level SDH variables used in the SDH-based risk adjustment models. For the nonracial subgroups, we provide the lowest decile threshold that was used to determine the vulnerable subgroups in all of the presented subgroup analyses in both the main text and Supplemental Information.

**Table S2. Demographic Statistics of the Data Subsets by Geographic Location**

|  | Training Set | | | Test Set | | |
| --- | --- | --- | --- | --- | --- | --- |
| Location | Members Total, No. | Female Total, No. (%) | Age, mean [median] (Std) | Members Total, No. | Female Total, No. (%) | Age, mean [median] (Std) |
| Alabama | 7004 | 3446 (49.2%) | 43.3 [45.0] (13.2) | 750 | 377 (50.3%) | 43.2 [45.0] (13.0) |
| Alaska | 845 | 383 (45.3%) | 39.8 [39.0] (12.9) | 89 | 38 (42.7%) | 40.8 [42.0] (12.4) |
| Arizona | 33633 | 16638 (49.5%) | 41.0 [41.0] (13.4) | 3773 | 1859 (49.3%) | 40.9 [40.0] (13.4) |
| Arkansas | 14279 | 7286 (51.0%) | 46.4 [49.0] (12.7) | 1537 | 786 (51.1%) | 46.5 [50.0] (12.9) |
| California | 109379 | 52834 (48.3%) | 39.9 [39.0] (12.6) | 12330 | 6078 (49.3%) | 39.9 [39.0] (12.6) |
| Colorado | 32085 | 15291 (47.7%) | 40.0 [38.0] (12.8) | 3579 | 1703 (47.6%) | 40.4 [39.0] (12.8) |
| Connecticut | 8061 | 3938 (48.9%) | 41.5 [42.0] (13.1) | 875 | 415 (47.4%) | 42.3 [44.0] (12.8) |
| Delaware | 1301 | 546 (42.0%) | 40.5 [40.0] (12.9) | 155 | 74 (47.7%) | 40.9 [41.0] (12.9) |
| District of Columbia | 3703 | 1807 (48.8%) | 37.3 [33.0] (11.9) | 394 | 202 (51.3%) | 36.9 [33.0] (11.2) |
| Florida | 75661 | 37752 (49.9%) | 41.6 [42.0] (13.1) | 8369 | 4038 (48.2%) | 41.7 [42.0] (13.0) |
| Georgia | 42475 | 22196 (52.3%) | 44.6 [47.0] (13.2) | 4714 | 2437 (51.7%) | 44.7 [47.0] (13.1) |
| Hawaii | 442 | 206 (46.6%) | 46.4 [49.0] (13.3) | 42 | 18 (42.9%) | 47.1 [50.5] (12.5) |
| Idaho | 2612 | 1273 (48.7%) | 40.7 [40.0] (13.2) | 315 | 159 (50.5%) | 40.5 [40.0] (13.6) |
| Illinois | 45286 | 21689 (47.9%) | 40.0 [39.0] (12.9) | 5044 | 2379 (47.2%) | 39.9 [39.0] (12.8) |
| Indiana | 30191 | 14503 (48.0%) | 42.1 [43.0] (13.3) | 3314 | 1664 (50.2%) | 42.0 [43.0] (13.4) |
| Iowa | 10296 | 5110 (49.6%) | 40.3 [40.0] (13.0) | 1203 | 600 (49.9%) | 40.1 [39.0] (13.1) |
| Kansas | 11622 | 5794 (49.9%) | 40.8 [40.0] (13.3) | 1290 | 664 (51.5%) | 40.9 [40.0] (13.0) |
| Kentucky | 9401 | 4460 (47.4%) | 40.5 [40.0] (12.7) | 1034 | 508 (49.1%) | 40.6 [41.0] (12.6) |
| Louisiana | 15414 | 7623 (49.5%) | 39.1 [38.0] (12.7) | 1620 | 802 (49.5%) | 39.1 [37.0] (12.9) |
| Maine | 1883 | 866 (46.0%) | 43.4 [45.0] (13.3) | 179 | 77 (43.0%) | 41.9 [43.0] (12.6) |
| Maryland | 20090 | 9721 (48.4%) | 39.7 [39.0] (12.5) | 2357 | 1163 (49.3%) | 39.8 [39.0] (12.6) |
| Massachusetts | 11796 | 5556 (47.1%) | 39.6 [38.0] (12.7) | 1343 | 627 (46.7%) | 40.4 [40.0] (13.1) |
| Michigan | 18491 | 8842 (47.8%) | 40.3 [40.0] (12.9) | 1965 | 943 (48.0%) | 40.7 [41.0] (13.0) |
| Minnesota | 25468 | 12351 (48.5%) | 39.5 [37.0] (12.8) | 2714 | 1318 (48.6%) | 39.7 [38.0] (12.8) |
| Mississippi | 11001 | 5347 (48.6%) | 40.5 [41.0] (13.0) | 1231 | 555 (45.1%) | 40.4 [41.0] (12.8) |
| Missouri | 30674 | 15420 (50.3%) | 43.4 [45.0] (13.4) | 3408 | 1713 (50.3%) | 43.5 [45.0] (13.5) |
| Montana | 1128 | 538 (47.7%) | 40.6 [40.0] (13.0) | 127 | 68 (53.5%) | 38.7 [38.0] (12.5) |
| Nebraska | 12153 | 5877 (48.4%) | 39.8 [38.0] (13.0) | 1331 | 628 (47.2%) | 38.9 [37.0] (13.2) |
| Nevada | 7039 | 3486 (49.5%) | 41.0 [41.0] (13.1) | 777 | 394 (50.7%) | 40.7 [40.0] (13.1) |
| New Hampshire | 2879 | 1383 (48.0%) | 42.2 [43.0] (13.4) | 332 | 151 (45.5%) | 42.6 [44.0] (13.0) |
| New Jersey | 19265 | 9407 (48.8%) | 40.0 [38.0] (12.7) | 2156 | 1078 (50.0%) | 39.6 [38.0] (12.7) |
| New Mexico | 5067 | 2361 (46.6%) | 40.5 [39.0] (13.3) | 554 | 258 (46.6%) | 40.6 [40.0] (13.5) |
| New York | 35343 | 17520 (49.6%) | 40.0 [37.0] (12.9) | 3900 | 1933 (49.6%) | 40.1 [38.0] (13.0) |
| North Carolina | 31466 | 15112 (48.0%) | 41.5 [42.0] (12.9) | 3583 | 1722 (48.1%) | 41.5 [42.0] (13.0) |
| North Dakota | 4088 | 1854 (45.4%) | 41.3 [41.0] (13.6) | 441 | 204 (46.3%) | 41.6 [41.0] (13.6) |
| Ohio | 36401 | 17487 (48.0%) | 40.7 [40.0] (13.3) | 4015 | 1941 (48.3%) | 40.6 [40.0] (13.2) |
| Oklahoma | 12692 | 5942 (46.8%) | 40.2 [39.0] (13.1) | 1425 | 666 (46.7%) | 39.8 [38.0] (12.9) |
| Oregon | 13217 | 6281 (47.5%) | 39.4 [38.0] (12.2) | 1447 | 665 (46.0%) | 39.6 [38.0] (11.9) |
| Pennsylvania | 29156 | 13759 (47.2%) | 41.0 [41.0] (12.9) | 3238 | 1509 (46.6%) | 41.1 [42.0] (12.9) |
| Puerto Rico | 56 | 20 (35.7%) | 37.4 [37.5] (11.8) | 6 | 6 (100.0%) | 43.2 [44.5] (5.3) |
| Rhode Island | 2869 | 1332 (46.4%) | 41.3 [41.0] (13.0) | 318 | 146 (45.9%) | 41.5 [40.5] (13.5) |
| South Carolina | 24220 | 13108 (54.1%) | 49.4 [53.0] (11.8) | 2699 | 1500 (55.6%) | 49.7 [53.0] (11.5) |
| South Dakota | 2219 | 1072 (48.3%) | 42.9 [44.0] (13.6) | 227 | 113 (49.8%) | 43.7 [45.0] (13.5) |
| Tennessee | 14495 | 6845 (47.2%) | 40.7 [40.0] (12.9) | 1667 | 742 (44.5%) | 40.3 [39.0] (12.9) |
| Texas | 133993 | 66500 (49.6%) | 40.5 [40.0] (13.0) | 14893 | 7387 (49.6%) | 40.6 [40.0] (13.0) |
| Utah | 13075 | 6367 (48.7%) | 37.8 [36.0] (12.6) | 1431 | 678 (47.4%) | 37.9 [36.0] (12.5) |
| Vermont | 539 | 275 (51.0%) | 46.5 [50.0] (12.2) | 63 | 24 (38.1%) | 47.9 [50.0] (13.1) |
| Virginia | 24881 | 12038 (48.4%) | 39.6 [38.0] (12.5) | 2742 | 1382 (50.4%) | 39.2 [38.0] (12.6) |
| Washington | 22264 | 10514 (47.2%) | 42.1 [42.0] (13.0) | 2455 | 1137 (46.3%) | 41.6 [42.0] (13.3) |
| West Virginia | 2331 | 1087 (46.6%) | 40.4 [41.0] (12.7) | 268 | 109 (40.7%) | 39.6 [39.0] (12.7) |
| Wisconsin | 31655 | 14909 (47.1%) | 42.0 [42.0] (13.5) | 3537 | 1652 (46.7%) | 41.5 [42.0] (13.4) |
| Wyoming | 1821 | 907 (49.8%) | 41.5 [42.0] (13.5) | 245 | 125 (51.0%) | 41.8 [43.0] (13.5) |

Demographic statistics of the administrative claims data for each of the data subsets. The training set was used to develop the risk adjustment models and the test set was used to evaluate out-of-sample performance of the models.

**Table S3. Variable Importances of the Prospective Linear Regression, LASSO Regression, Random Forest, and LightGBM Models without SDH Indicators**

| Linear Regression (Coef^a^) | LASSO (Coef^a^) | Random Forest (MDI^b^) | LightGBM (Gain^c^) |
| --- | --- | --- | --- |
| Male 25-35 (-5628.44) | Male 25-35 (-1883.26) | Chronic kidney disease (0.36) | Chronic kidney disease (227) |
| Male 18-25 (-5535.97) | Male 60-65 (1821.48) | Other aftercare (0.09) | Deficiency and other anemia (161) |
| Male 35-45 (-5115.62) | Male 18-25 (-1643.14) | Residual codes; unclassified (0.04) | Other aftercare (128) |
| Female 18-25 (-4885.79) | Chronic kidney disease (1378.90) | Fluid and electrolyte disorders (0.03) | Residual codes; unclassified (108) |
| Female 35-45 (-3763.26) | Male 35-45 (-1356.80) | Deficiency and other anemia (0.02) | Fluid and electrolyte disorders (99) |
| Male 45-55 (-3739.92) | OB-related trauma to perineum and vulva (-1299.17) | Secondary malignancies (0.02) | Other nervous system disorders (96) |
| Female 25-35 (-3482.14) | Male 55-60 (995.75) | Other nervous system disorders (0.02) | Hypertension with complications and secondary hypertension (96) |
| Female 45-55 (-3265.61) | Melanomas of skin (980.72) | Maintenance chemotherapy; radiotherapy (0.01) | Acute and unspecified renal failure (90) |
| Female 55-60 (-2667.59) | Suicide and intentional self-inflicted injury (973.93) | Essential hypertension (0.01) | Diabetes mellitus with complications (86) |
| Male 55-60 (-2290.72) | Female 18-25 (-964.31) | Other lower respiratory disease (0.01) | Essential hypertension (81) |
| Female 60-65 (-2278.73) | Other diseases of kidney and ureters (939.90) | Immunizations and screening for infectious disease (0.01) | Maintenance chemotherapy; radiotherapy (69) |
| OB-related trauma to perineum and vulva (-1977.64) | Female 60-65 (898.54) | Spondylosis; intervertebral disc disorders; other back problems (0.01) | Other lower respiratory disease (68) |
| Chronic kidney disease (1378.82) | Secondary malignancies (796.27) | Diabetes mellitus with complications (0.01) | Secondary malignancies (66) |
| Male 60-65 (-1359.50) | Lymphadenitis (792.32) | Congestive heart failure; nonhypertensive (0.01) | Congestive heart failure; nonhypertensive (63) |
| Suicide and intentional self-inflicted injury (1046.59) | Regional enteritis and ulcerative colitis (751.91) | Immunity disorders (0.01) | Other nutritional; endocrine; and metabolic disorders (62) |
| Melanomas of skin (1044.03) | Sickle cell anemia (723.33) | Other gastrointestinal disorders (0.01) | Spondylosis; intervertebral disc disorders; other back problems (62) |
| Other diseases of kidney and ureters (948.70) | Multiple sclerosis (722.04) | Nonspecific chest pain (0.01) | Other diseases of kidney and ureters (61) |
| Lymphadenitis (874.83) | Complication of device; implant or graft (610.38) | Substance-related disorders (0.01) | Chronic ulcer of skin (58) |
| Secondary malignancies (793.36) | Female 55-60 (606.10) | Other nutritional; endocrine; and metabolic disorders (0.01) | Other gastrointestinal disorders (57) |
| Regional enteritis and ulcerative colitis (762.89) | External cause codes: Fall (576.08) | Other connective tissue disease (0.01) | Substance-related disorders (56) |

^a^Coefficient for the traditional and penalized linear regression models. Positive value means higher predicted cost for higher value of the predictor, and negative value means lower predicted cost for higher value of the predictor. The intercepts of the linear and LASSO models were 6761.90 and 3142.32 respectively.

^b^MDI (mean decrease impurity) is the sum over the number of splits (across all trees) that use the predictor, proportional to the number of samples it splits.

^c^Gain represents the relative contribution of the predictor, measured by the extent of improvement in prediction after adding the split on the predictor. Gain values are all non-negative.

Variable importances of the prospective linear regression model, LASSO regression model, random forest model, and LightGBM model trained without SDH indicators. ­­

**Table S4. Variable Importances of the Prospective Linear Regression, LASSO Regression, Random Forest, and LightGBM Models with SDH Indicators**

| Linear Regression(Coef^a^) | LASSO (Coef^a^) | Random Forest (MDI^b^) | LightGBM (Gain^c^) |
| --- | --- | --- | --- |
| Population Without Health Insurance Coverage (3925.92) | Population Without Health Insurance Coverage (4551.98) | Chronic kidney disease (0.32) | Chronic kidney disease (224) |
| Male 25-35 (-2410.88) | Male 60-65 (2153.25) | Other aftercare (0.08) | Deficiency and other anemia (183) |
| Male 18-25 (-2357.83) | Male 25-35 (-1898.68) | Residual codes; unclassified (0.03) | Other aftercare (143) |
| Population Speak English Less than "Very Well" (-2264.26) | Male 18-25 (-1760.41) | Fluid and electrolyte disorders (0.03) | Diabetes mellitus with complications (116) |
| OB-related trauma to perineum and vulva (-2095.68) | OB-related trauma to perineum and vulva (-1717.27) | Deficiency and other anemia (0.02) | Other nervous system disorders (115) |
| Population Asian (-1976.94) | Male 35-45 (-1379.83) | Secondary malignancies (0.02) | Essential hypertension (105) |
| Population White (-1938.60) | Chronic kidney disease (1379.34) | Other nervous system disorders (0.01) | Other lower respiratory disease (102) |
| Male 35-45 (-1823.35) | Male 55-60 (1267.21) | Maintenance chemotherapy; radiotherapy (0.01) | Spondylosis; intervertebral disc disorders; other back problems (98) |
| Male 60-65 (1714.00) | Female 60-65 (1218.64) | Other lower respiratory disease (0.01) | Fluid and electrolyte disorders (95) |
| Female 18-25 (-1623.73) | Female 18-25 (-1103.24) | Essential hypertension (0.01) | Acute and unspecified renal failure (95) |
| Population Obtained High School Diploma (-1459.17) | Melanomas of skin (1024.41) | Immunizations and screening for infectious disease (0.01) | Hypertension with complications and secondary hypertension (93) |
| Chronic kidney disease (1378.91) | Suicide and intentional self-inflicted injury (1021.09) | Spondylosis; intervertebral disc disorders; other back problems (0.01) | Residual codes; unclassified (93) |
| Gini Index of Income Inequality (-1317.88) | Other diseases of kidney and ureters (944.43) | Population American Indian and Alaska Native (0.01) | Congestive heart failure; nonhypertensive (77) |
| Families with Single Parent (-1194.94) | Female 55-60 (877.34) | Population Asian (0.01) | Other gastrointestinal disorders (76) |
| Families Received Food Stamps/Snap in the Past 12 months (-1151.06) | Population African American (869.78) | Congestive heart failure; nonhypertensive (0.01) | Maintenance chemotherapy; radiotherapy (75) |
| Melanomas of skin (1069.73) | Lymphadenitis (846.38) | Gini Index of Income Inequality (0.01) | Other diseases of kidney and ureters (72) |
| Suicide and intentional self-inflicted injury (1033.38) | Secondary malignancies (794.57) | Population Without Health Insurance Coverage (0.01) | Other nutritional; endocrine; and metabolic disorders (70) |
| Other diseases of kidney and ureters (946.92) | Regional enteritis and ulcerative colitis (759.65) | Immunity disorders (0.01) | Nausea and vomiting (69) |
| Male 55-60 (941.10) | Sickle cell anemia (731.96) | Diabetes mellitus with complications (0.01) | Secondary malignancies (65) |
| Population American Indian and Alaska Native (-882.69) | Multiple sclerosis (728.73) | Population Obtained High School Diploma (0.01) | Chronic ulcer of skin (59) |

^a^Coefficient for the traditional and penalized linear regression models. Positive value means higher predicted cost for higher value of the predictor, and negative value means lower predicted cost for higher value of the predictor. The intercepts of the linear and LASSO models respectively were 6761.90 and 2756.91.

^b^MDI (mean decrease impurity) is the sum over the number of splits (across all trees) that use the predictor, proportional to the number of samples it splits.

^c^Gain represents the relative contribution of the predictor, measured by the extent of improvement in prediction after adding the split on the predictor. Gain values are all non-negative.

Variable importances of the prospective linear regression model, LASSO regression model, random forest model, and LightGBM model trained with ZIP code-level SDH indicators.

**Table S5. Performance Measures of LASSO Regression on the Test Set**

| Evaluation Metric | No SDH | SDH |
| --- | --- | --- |
| R^2^ (95% CI)^a^ |  |  |
| Linear | 0.327 (0.300, 0.353) | 0.327 (0.300, 0.354) |
| LASSO | 0.327 (0.300, 0.353) | 0.327 (0.300, 0.354) |
| MLP | 0.358 (0.328, 0.388) | 0.369 (0.339, 0.401) |
| MLP with LASSO | 0.361 (0.329, 0.393) | 0.369 (0.339, 0.400) |
| Random Forest | 0.377 (0.345, 0.409) | 0.372 (0.340, 0.404) |
| LightGBM | 0.388 (0.357, 0.420) | 0.387 (0.357, 0.419) |
| MAE (95% CI)^b^ |  |  |
| Linear | 6992 (6889, 7094) | 6991 (6889, 7094) |
| LASSO | 7001 (6898, 7104) | 6992 (6889, 7094) |
| MLP | 6826 (6725, 6926) | 6806 (6707, 6906) |
| MLP with LASSO | 6681 (6581, 6782) | 6804 (6705, 6904) |
| Random Forest | 6791 (6692, 6890) | 6832 (6732, 6931) |
| LightGBM | 6637 (6539, 6735) | 6634 (6536, 6732) |
| C-statistic (95% CI)^c^ |  |  |
| Linear | 0.703 (0.701, 0.705) | 0.700 (0.699, 0.702) |
| LASSO | 0.706 (0.704, 0.708) | 0.702 (0.701, 0.704) |
| MLP | 0.711 (0.709, 0.713) | 0.712 (0.710, 0.713) |
| MLP with LASSO | 0.712 (0.711, 0.714) | 0.706 (0.704, 0.708) |
| Random Forest | 0.706 (0.705, 0.708) | 0.705 (0.703, 0.706) |
| ML | 0.717 (0.715, 0.718) | 0.716 (0.714, 0.717) |

Abbreviations: MLP, Multilayer Perceptron;

MAE, Mean Absolute Error;

R^2^, Coefficient of Determination;

SDH, Social Determinants of Health

Comparison of performance measures between traditional linear regression, penalized linear regression (LASSO), multilayer perceptron developed using all predictors and using LASSO-selected predictors, random forest, and LightGBM prospective models, predicting 2017 yearly top-coded spending from 2016 characteristics. The SDH model additionally includes 17 ZIP code-level SDH variables obtained from U.S. Census data.

^a^Confidence intervals for R^2^ were constructed using the nonparametric bootstrap.^13^

^b^Confidence intervals for MAE were constructed using a paired t-test.

^c^Confidence intervals for C-statistic were constructed using a jackknife procedure.^14^

**Table S6. Predictive Ratio and Net Compensation Values of Prospective Machine Learning Models on Age and Sex Subgroups in the Test Set**

|  |  |  | Model Predictive Ratio^b^ and  Net Compensation^c^ | |
| --- | --- | --- | --- | --- |
| Subgroup | No. (%) | 2017 Spending ($)^a^ | ML (95% CI) | ML with SDH (95% CI) |
| Total | 117616 (100.0) | 6677 | 1.000 (0.976, 1.024) | 1.000 (0.976, 1.024) |
|  |  |  | 0 (-105, 105) | 0 (-105, 105) |
| Female, age 18-24 | 6701 (5.7) | 3707 | 0.937 (0.853, 1.021) | 0.940 (0.855, 1.025) |
|  |  |  | 249 (-23, 520) | 236 (-31, 503) |
| Female, age 25-34 | 13972 (11.9) | 5749 | 1.045 (0.995, 1.095) | 1.041 (0.991, 1.092) |
|  |  |  | -248 (-471, -24) | -228 (-451, -6) |
| Female, age 35-44 | 11583 (9.8) | 6693 | 1.010 (0.943, 1.078) | 1.012 (0.945, 1.080) |
|  |  |  | -69 (-371, 233) | -82 (-382, 219) |
| Female, age 45-54 | 12562 (10.7) | 9203 | 1.021 (0.958, 1.085) | 1.016 (0.953, 1.080) |
|  |  |  | -193 (-584, 199) | -147 (-537, 243) |
| Female, age 55-59 | 7196 (6.1) | 12214 | 1.023 (0.944, 1.102) | 1.014 (0.936, 1.093) |
|  |  |  | -278 (-893, 337) | -174 (-784, 436) |
| Female, age 60-64 | 5455 (4.6) | 14009 | 0.986 (0.904, 1.069) | 0.980 (0.898, 1.061) |
|  |  |  | 195 (-521, 912) | 291 (-420, 1001) |
| Male, age 18-24 | 7336 (6.2) | 2157 | 0.857 (0.752, 0.963) | 0.864 (0.759, 0.970) |
|  |  |  | 359 (154, 564) | 339 (126, 552) |
| Male, age 25-34 | 15465 (13.1) | 2114 | 0.932 (0.859, 1.005) | 0.958 (0.884, 1.033) |
|  |  |  | 153 (18, 289) | 92 (-45, 228) |
| Male, age 35-44 | 12434 (10.6) | 3287 | 0.906 (0.826, 0.987) | 0.916 (0.835, 0.998) |
|  |  |  | 340 (138, 542) | 300 (98, 502) |
| Male, age 45-54 | 12672 (10.8) | 6879 | 0.971 (0.895, 1.047) | 0.986 (0.909, 1.063) |
|  |  |  | 206 (-139, 551) | 98 (-255, 451) |
| Male, age 55-59 | 7020 (6.0) | 10634 | 1.042 (0.950, 1.135) | 1.035 (0.943, 1.127) |
|  |  |  | -432 (-1032, 169) | -361 (-963, 240) |
| Male, age 60-64 | 5220 (4.4) | 13694 | 1.042 (0.946, 1.138) | 1.033 (0.938, 1.128) |
|  |  |  | -549 (-1380, 281) | -434 (-1266, 399) |

Abbreviations: ML, Machine Learning;

SDH, Social Determinants of Health

Comparison of machine learning-based prospective risk adjustment models without and with the addition of ZIP code-level SDH indicators as predictors (see Table 1 in the main text for a complete list of SDH indicators) on the age and sex subgroups. The predictions for each model were adjusted so that the mean of the predictions over the total test population was equal to the mean of the actual costs, resulting in a predictive ratio of exactly 1.0 over the total test set population. Subgroups were composed of members in the lowest decile of ZIP codes with respect to the corresponding SDH variable (see **Table S1**).

^a^Spending included all healthcare utilization in 2017 of members with full enrollment in 2016 and 2017. Values larger than $400,000 were replaced with $400,000.

^b^Predictive ratio for a subgroup was computed as the ratio of the mean of observed to the mean of predicted spending over the subgroup. Approximate confidence intervals for predictive ratios were computed with the delta method.^24^

^c^Net compensation for a subgroup was computed as the mean difference between predicted and observed spending in the subgroup. Confidence intervals were estimated using a paired t-test.

**Table S7. Predictive Ratio and Net Compensation Values of Prospective Linear Models on SDH-Based Subgroups in the Test Set**

|  |  |  | Model Predictive Ratio**^b^** and  Net Compensation^c^ | |
| --- | --- | --- | --- | --- |
| Subgroup | No. (%) | 2017 Spending ($)^a^ | Linear (95% CI) | Linear with SDH (95% CI) |
| Total | 117616 (100.0) | 6677 | 1.000 (0.977, 1.023) | 1.000 (0.977, 1.023) |
|  |  |  | 0 (-110, 110) | 0 (-110, 110) |
| Poverty |  |  |  |  |
| Median Income in the Past 12 Months, $ | 4923 (4.2) | 10818 | 1.092 (0.988, 1.195) | 1.054 (0.955, 1.153) |
|  |  |  | -907 (-1633, -182) | -556 (-1281, 170) |
| Families Under 0.5 Ratio of Income to Poverty Level in the Past 12 Months, % | 7932 (6.7) | 9344 | 1.014 (0.929, 1.098) | 0.968 (0.888, 1.047) |
|  |  |  | -127 (-650, 396) | 313 (-210, 836) |
| Families Between 0.5 and 0.74 Ratio of Income to Poverty Level in the Past 12 Months, % | 6651 (5.7) | 8952 | 1.065 (0.969, 1.161) | 1.024 (0.933, 1.116) |
|  |  |  | -543 (-1117, 31) | -211 (-785, 363) |
| Families Between 0.75 and 0.99 Ratio of Income to Poverty Level in the Past 12 Months, % | 7194 (6.1) | 9395 | 1.094 (1.000, 1.187) | 1.062 (0.971, 1.152) |
|  |  |  | -805 (-1373, -237) | -545 (-1112, 23) |
| Families Received Food Stamps/Snap in the Past 12 months, % | 9009 (7.7) | 9001 | 1.069 (0.984, 1.155) | 1.032 (0.950, 1.114) |
|  |  |  | -584 (-1067, -101) | -277 (-759, 206) |
| Population Unemployed, % | 10278 (8.7) | 7055 | 0.959 (0.888, 1.030) | 0.923 (0.855, 0.990) |
|  |  |  | 300 (-85, 686) | 592 (207, 977) |
| Gini Index of Income Inequality | 16155 (13.7) | 6138 | 1.028 (0.964, 1.093) | 1.031 (0.967, 1.096) |
|  |  |  | -170 (-450, 110) | -187 (-467, 93) |
| Education |  |  |  |  |
| Population Obtained High School Diploma, % | 9482 (8.1) | 7555 | 1.012 (0.928, 1.095) | 0.970 (0.891, 1.050) |
|  |  |  | -89 (-533, 356) | 231 (-214, 676) |
| Population Obtained Bachelor's Degree, % | 4169 (3.5) | 11338 | 1.092 (0.983, 1.201) | 1.066 (0.960, 1.171) |
|  |  |  | -955 (-1800, -111) | -700 (-1545, 144) |
| Other |  |  |  |  |
| Population Speak English Less than "Very Well", % | 23659 (20.1) | 5453 | 0.984 (0.930, 1.039) | 1.003 (0.947, 1.058) |
|  |  |  | 88 (-141, 316) | -14 (-243, 214) |
| Families with Single Parent, % | 9097 (7.7) | 9880 | 1.064 (0.981, 1.147) | 1.014 (0.936, 1.093) |
|  |  |  | -592 (-1125, -60) | -141 (-673, 391) |
| Population Without Health Insurance Coverage, % | 13656 (11.6) | 8333 | 1.096 (1.023, 1.170) | 1.044 (0.974, 1.113) |
|  |  |  | -733 (-1115, -351) | -348 (-730, 33) |

Abbreviations: SDH, Social Determinants of Health

Comparison of linear regression-based prospective risk adjustment models without and with the addition of ZIP code-level SDH indicators as predictors (see Table 1 in the main text for a complete list of SDH indicators) on the socioeconomic subgroups. The predictions for each model were adjusted so that the mean of the predictions over the total test population was equal to the mean of the actual costs, resulting in a predictive ratio of exactly 1.0 over the total test set population. Subgroups were composed of members in the lowest decile of ZIP codes with respect to the corresponding SDH variable (see **Table S1**).

^a^Spending included all healthcare utilization in 2017 of members with full enrollment in 2016 and 2017. Values larger than $400,000 were replaced with $400,000.

^b^Predictive ratio for a subgroup was computed as the ratio of the mean of observed to the mean of predicted spending over the subgroup. Approximate confidence intervals for predictive ratios were computed with the delta method.^24^

^c^Net compensation for a subgroup was computed as the mean difference between predicted and observed spending in the subgroup. Confidence intervals were estimated using a paired t-test.

**Table S8. Predictive Ratio and Net Compensation Values of Prospective Linear Models on Age and Sex Subgroups in the Test Set**

|  |  |  | Model Predictive Ratio**^b^** and  Net Compensation^c^ | |
| --- | --- | --- | --- | --- |
| Subgroup | No. (%) | 2017 Spending ($)^a^ | Linear (95% CI) | Linear with SDH (95% CI) |
| Total | 117616 (100.0) | 6677 | 1.000 (0.977, 1.023) | 1.000 (0.977, 1.023) |
|  |  |  | 0 (-110, 110) | 0 (-110, 110) |
| Female, age 18-24 | 6701 (5.7) | 3707 | 1.012 (0.922, 1.101) | 0.999 (0.910, 1.087) |
|  |  |  | -43 (-333, 247) | 5 (-284, 295) |
| Female, age 25-34 | 13972 (11.9) | 5749 | 1.016 (0.969, 1.063) | 1.014 (0.968, 1.061) |
|  |  |  | -93 (-322, 137) | -82 (-311, 147) |
| Female, age 35-44 | 11583 (9.8) | 6693 | 1.018 (0.955, 1.082) | 1.022 (0.958, 1.086) |
|  |  |  | -121 (-436, 194) | -143 (-458, 171) |
| Female, age 45-54 | 12562 (10.7) | 9203 | 1.002 (0.941, 1.063) | 1.003 (0.942, 1.064) |
|  |  |  | -21 (-424, 382) | -29 (-432, 374) |
| Female, age 55-59 | 7196 (6.1) | 12214 | 1.001 (0.928, 1.074) | 0.998 (0.925, 1.071) |
|  |  |  | -13 (-647, 621) | 23 (-611, 657) |
| Female, age 60-64 | 5455 (4.6) | 14009 | 0.971 (0.894, 1.049) | 0.976 (0.898, 1.054) |
|  |  |  | 413 (-354, 1181) | 341 (-426, 1108) |
| Male, age 18-24 | 7336 (6.2) | 2157 | 0.964 (0.844, 1.084) | 0.980 (0.858, 1.102) |
|  |  |  | 81 (-129, 291) | 44 (-166, 254) |
| Male, age 25-34 | 15465 (13.1) | 2114 | 1.021 (0.944, 1.099) | 1.033 (0.955, 1.112) |
|  |  |  | -44 (-183, 95) | -68 (-208, 71) |
| Male, age 35-44 | 12434 (10.6) | 3287 | 0.944 (0.861, 1.027) | 0.928 (0.846, 1.009) |
|  |  |  | 196 (-8, 399) | 256 (52, 459) |
| Male, age 45-54 | 12672 (10.8) | 6879 | 0.999 (0.924, 1.073) | 0.994 (0.919, 1.068) |
|  |  |  | 10 (-367, 388) | 44 (-333, 422) |
| Male, age 55-59 | 7020 (6.0) | 10634 | 1.011 (0.927, 1.096) | 1.009 (0.925, 1.093) |
|  |  |  | -118 (-758, 522) | -95 (-734, 545) |
| Male, age 60-64 | 5220 (4.4) | 13694 | 1.008 (0.921, 1.095) | 1.017 (0.930, 1.105) |
|  |  |  | -109 (-981, 764) | -235 (-1107, 636) |

Abbreviations: SDH, Social Determinants of Health

Comparison of linear regression-based prospective risk adjustment models without and with the addition of ZIP code-level SDH indicators as predictors (see Table 1 in the main text for a complete list of SDH indicators) on the age and sex subgroups. The predictions for each model were adjusted so that the mean of the predictions over the total test population was equal to the mean of the actual costs, resulting in a predictive ratio of exactly 1.0 over the total test set population.

^a^Spending included all healthcare utilization in 2017 of members with full enrollment in 2016 and 2017. Values larger than $400,000 were replaced with $400,000.

^b^Predictive ratio for a subgroup was computed as the ratio of the mean of observed to the mean of predicted spending over the subgroup. Approximate confidence intervals for predictive ratios were computed with the delta method.^24^

^c^Net compensation for a subgroup was computed as the mean difference between predicted and observed spending in the subgroup. Confidence intervals were estimated using a paired t-test.

**Figure S1. Binned Scatter Plots of the Prospective Linear Regression and Machine Learning Models without and with SDH Indicators on the Test Set**


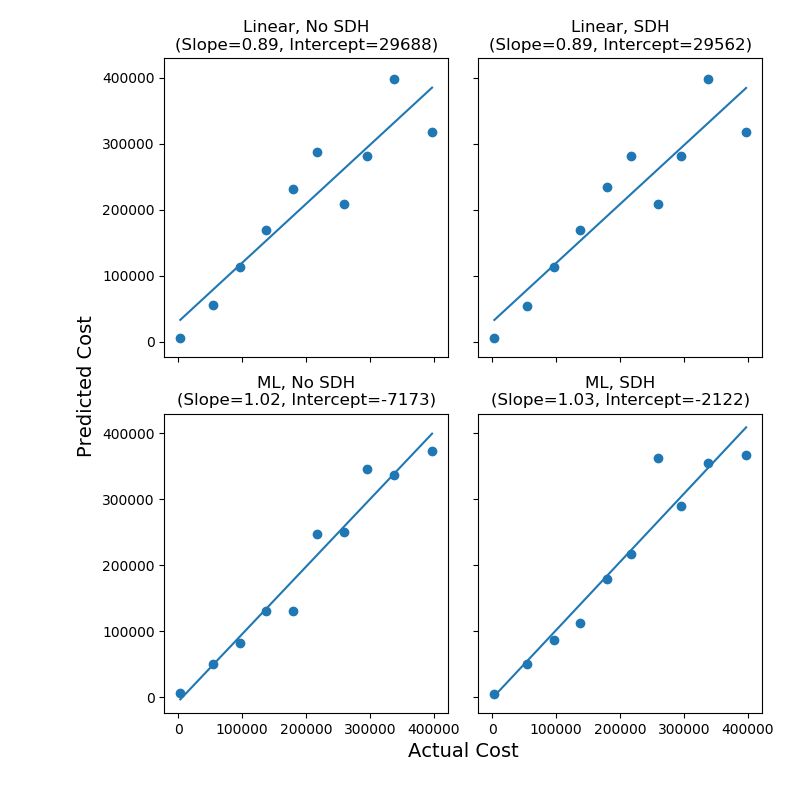


Abbreviations: ML, Machine Learning;

SDH, Social Determinants of Health

Actual versus predicted cost scatter plots of traditional linear regression machine learning prospective models, predicting 2017 yearly top-coded spending from 2016 characteristics. The SDH models additionally include 17 ZIP code-level SDH variables obtained from U.S. Census data. The predictions and actual costs were each divided into 10 equal bins, and the mean of each bin is plotted. A regression line fit on the binned values is plotted with slope and intercept shown in the title of each subplot.

**Table S9. Performance Measures of Models Derived Using Binary Diagnosis Predictors on the Test Set**

| Evaluation Metric | CCS Binary Diagnosis Variables, No SDH | CCS Binary Diagnosis Variables, SDH | CCS Count Diagnosis Variables, No SDH | CCS Count Diagnosis Variables, SDH |
| --- | --- | --- | --- | --- |
| R^2^ (95% CI)^a^ |  |  |  |  |
| Linear | 0.231 (0.215, 0.248) | 0.231 (0.215, 0.248) | 0.327 (0.300, 0.353) | 0.327 (0.300, 0.354) |
| ML | 0.314 (0.285, 0.343) | 0.311 (0.282, 0.340) | 0.388 (0.357, 0.420) | 0.387 (0.357, 0.419) |
| MAE (95% CI)^b^ |  |  |  |  |
| Linear | 7199 (7088, 7309) | 7207 (7096, 7317) | 6992 (6889, 7094) | 6991 (6889, 7094) |
| ML | 6888 (6784, 6992) | 6913 (6808, 7017) | 6637 (6539, 6735) | 6634 (6536, 6732) |
| C-statistic (95% CI)^c^ |  |  |  |  |
| Linear | 0.698 (0.696, 0.699) | 0.695 (0.693, 0.697) | 0.703 (0.701, 0.705) | 0.700 (0.699, 0.702) |
| ML | 0.713 (0.711, 0.715) | 0.707 (0.706, 0.709) | 0.717 (0.715, 0.718) | 0.716 (0.714, 0.717) |

Abbreviations: ML, Machine Learning;

MAE, Mean Absolute Error;

R^2^, Coefficient of Determination;

MS-DRG, Medicare Severity Diagnosis Related Groups;

HCC, Hierarchical Condition Categories;

SDH, Social Determinants of Health

Comparison of performance measures between linear regression and machine learning prospective risk adjustment models, predicting 2017 yearly top-coded spending from 2016 characteristics. The CCS binary model encoded diagnostic predictors as binary indicators and the CCS count model encoded diagnostic predictors as counts over the year (results duplicated from main text). The SDH model additionally includes 17 ZIP code-level SDH variables obtained from U.S. Census data.

^a^Confidence intervals for R^2^ were constructed using the nonparametric bootstrap.^25^

^b^Confidence intervals for MAE were constructed using a paired t-test.

^c^Confidence intervals for C-statistic were constructed using a jackknife procedure.^26^

**Table S10. Performance Measures of Top-Coded and Non-Top-Coded Models on the Test Set**

| Evaluation Metric | Top-Coded at $400,000 | Not Top-Coded |
| --- | --- | --- |
| R^2^ (95% CI)^a^ |  |  |
| Linear | 0.327 (0.300, 0.354) | 0.320 (0.281, 0.358) |
| ML | 0.387 (0.357, 0.419) | 0.387 (0.352, 0.423) |
| MAE (95% CI)^b^ |  |  |
| Linear | 6991 (6889, 7094) | 7288 (7164, 7412) |
| ML | 6634 (6536, 6732) | 6937 (6819, 7055) |
| C-statistic (95% CI)^c^ |  |  |
| Linear | 0.700 (0.699, 0.702) | 0.700 (0.699, 0.702) |
| ML | 0.716 (0.714, 0.717) | 0.716 (0.714, 0.717) |

Abbreviations: ML, Machine Learning;

MAE, Mean Absolute Error;

R^2^, Coefficient of Determination;

SDH, Social Determinants of Health

Comparison of performance measures between linear regression and machine learning prospective risk adjustment models without and with top-coding predicted and observed spending values to $400,000 (values larger than $400,000 were clipped to $400,000 in the top-coded model, and not modified in the non-top-coded model). All models were trained with the addition of ZIP code-level SDH indicator variables.

^a^Confidence intervals for R^2^ were constructed using the nonparametric bootstrap.^25^

^b^Confidence intervals for MAE were constructed using a paired t-test.

^c^Confidence intervals for C-statistic were constructed using a jackknife procedure.^26^

**Table S11. Performance Measures of Models with Lab Results on the Test Set**

| Evaluation Metric | No Lab Results | Lab Results |
| --- | --- | --- |
| R^2^ (95% CI)^a^ |  |  |
| Linear | 0.327 (0.300, 0.354) | 0.331 (0.304, 0.358) |
| ML | 0.387 (0.357, 0.419) | 0.383 (0.352, 0.414) |
| MAE (95% CI)^b^ |  |  |
| Linear | 6991 (6889, 7094) | 6972 (6870, 7075) |
| ML | 6634 (6536, 6732) | 6640 (6542, 6739) |
| C-statistic (95% CI)^c^ |  |  |
| Linear | 0.700 (0.699, 0.702) | 0.695 (0.694, 0.697) |
| ML | 0.716 (0.714, 0.717) | 0.715 (0.714, 0.717) |

Abbreviations: ML, Machine Learning;

MAE, Mean Absolute Error;

R^2^, Coefficient of Determination;

SDH, Social Determinants of Health

Comparison of performance measures between linear regression and machine learning prospective risk adjustment models without and with the inclusion of lab test results as predictors. All models were trained with the addition of ZIP code-level SDH indicator variables.

^a^Confidence intervals for R^2^ were constructed using the nonparametric bootstrap.^25^

^b^Confidence intervals for MAE were constructed using a paired t-test.

^c^Confidence intervals for C-statistic were constructed using a jackknife procedure.^26^

**Table S12. Performance Measures of Concurrent and Prospective Models with SDH Indicators on the Test Set**

|  | Concurrent Model Evaluated Concurrently | Concurrent Model Evaluated Prospectively | Prospective Model Evaluated Prospectively |
| --- | --- | --- | --- |
| R^2^ (95% CI)^a^ |  |  |  |
| Linear | 0.656 (0.635, 0.676) | 0.240 (0.209, 0.272) | 0.327 (0.300, 0.354) |
| ML | 0.733 (0.713, 0.752) | 0.276 (0.240, 0.312) | 0.387 (0.357, 0.419) |
| MAE (95% CI)^b^ |  |  |  |
| Linear | 3560 (3493, 3627) | 7097 (6987, 7207) | 6991 (6889, 7094) |
| ML | 3115 (3056, 3174) | 7031 (6923, 7138) | 6634 (6536, 6732) |
| C-statistic (95% CI)^c^ |  |  |  |
| Linear | 0.847 (0.846, 0.848) | 0.688 (0.686, 0.689) | 0.700 (0.699, 0.702) |
| ML | 0.865 (0.864, 0.866) | 0.695 (0.693, 0.697) | 0.716 (0.714, 0.717) |

Abbreviations: ML, Machine Learning;

MAE, Mean Absolute Error;

R^2^, Coefficient of Determination;

SDH, Social Determinants of Health

Comparison of performance measures between a model trained in the concurrent frame evaluated concurrently and prospectively, in addition to the model trained in the prospective frame evaluated prospectively. All models were trained with the addition of ZIP code-level SDH indicator variables.

^a^Confidence intervals for R^2^ were constructed using the nonparametric bootstrap.^25^

^b^Confidence intervals for MAE were constructed using a paired t-test.

^c^Confidence intervals for C-statistic were constructed using a jackknife procedure.^26^
